# Early Driving Pressure Changes Predict Outcomes during Venovenous Extracorporeal Membrane Oxygenation for Acute Respiratory Distress Syndrome

**DOI:** 10.1155/2020/6958152

**Published:** 2020-03-07

**Authors:** Harry Magunia, Helene A. Haeberle, Philipp Henn, Martin Mehrländer, Peer O. Vlatten, Valbona Mirakaj, Peter Rosenberger, Michael Koeppen

**Affiliations:** Department of Anesthesiology and Intensive Care Medicine, University Hospital, Tübingen, Germany

## Abstract

**Background:**

Extracorporeal membrane oxygenation (ECMO) serves as a rescue therapy when systemic hypoxia persists despite conventional care for severe acute respiratory distress syndrome (ARDS). Due to the extracorporeal gas exchange, the *p*_a_O_2_/*F*_i_O_2_ ratio cannot be used as the primary marker for disease severity and progression. Therefore, we performed a propensity score-matched analysis to identify other potential predictors of outcomes in patients supported by ECMO therapy.

**Results:**

Between December 2014 and May 2018, 105 patients underwent venovenous ECMO in our institution. From these patients, we identified 28 who died during ECMO therapy and assigned 28 control patients using propensity score matching based on the following criteria: age, ARDS severity, and SAPSII score at admission. A statistical evaluation of the patient characteristics, intensive care data, morbidities, respiratory system variables, and outcomes was performed. The baseline patient characteristics did not differ between groups and ECMO was placed on day 1 in all patients. The analyzed variables of respiratory mechanics, such as the plateau pressure, positive end-expiratory pressure, and tidal volume, did not differ between groups. The driving pressure before ECMO was equal between the nonsurvivors and the controls. Twelve hours after initiation of ECMO therapy, the driving pressure decreased by 40.8% in the survivors but by only 20.1% in the nonsurvivors.

**Conclusions:**

We report that very early driving pressure changes can serve as an indicator of disease severity and predict patient survival following ECMO therapy.

## 1. Background

Acute respiratory distress syndrome (ARDS) develops from acute pneumonia, nonpulmonary sepsis, aspiration of gastric content, and major trauma. All of these pathologies induce severe inflammation of the lung that becomes clinically apparent as systemic hypoxia due to impairment of pulmonary gas exchange. The clinical correlates of this inflammatory process have been defined using three categories of severity of ARDS—mild, moderate, and severe—depending on the oxygenation level [1]. Since the initial description of ARDS by Ashbaugh in 1967 [2], considerable progress has been made in therapy through optimizing mechanical ventilation and supportive care. However, because mechanical ventilation may lead to additional damage to an already injured lung, optimizing ventilation strategies for use in ARDS patients is an important goal [3].

In cases where the sensitive equilibrium between safe ventilation and oxygenation is imbalanced, extracorporeal membrane oxygenation (ECMO) can ensure oxygenation and decarboxylation until the lung heals. EMCO can lead to less invasive mechanical ventilation and reduces the risk of additional damage to the injured lungs. This risk reduction is achieved by establishing an extracorporeal circuit for venovenous ECMO (VV-ECMO). The use of EMCO has steadily increased recently [4], but the role of ECMO in ARDS treatment remains under discussion. Randomized controlled trials have demonstrated a survival benefit [5], but other reports have shown indifferent effects [6]. Recently, a retrospective study found that ECMO treatment for ARDS improved the health-related quality of life after 1 year [7].

After ECMO initiation, the value of the *p*aO_2_/*F*iO_2_ ratio measurement as a parameter of pulmonary gas exchange is lost. We hypothesized that variables associated with pulmonary mechanics during invasive ventilation might correlate with lung injury and potentially with mortality and thus could be used in place of the *p*aO_2_/*F*iO_2_ ratio. To test this hypothesis, we performed a propensity score-matched analysis of variables describing respiratory mechanics at 12 hours after ECMO initiation in survivors and nonsurvivors to identify a very early marker of disease severity.

## 2. Methods

### 2.1. Study Population and Ethical Approval

All data were collected retrospectively from the medical records of the University Hospital of Tübingen. The study was approved by the ethics committee of the University Hospital Tübingen (768/2018BO2), which waived the need for informed consent, because patient anonymity was maintained. All the methods were approved by the local IRB and performed in accordance with the Declaration of Helsinki and the relevant guidelines. All patients treated with ECMO for ARDS at the Department for Anesthesiology and Intensive Care Medicine between December 2014 and May 2018 were retrospectively screened for inclusion into this observational cohort study.

### 2.2. VV-ECMO Indication and Placement

Patients were admitted to our institution either directly or via secondary transfer from other hospitals for ARDS treatment. The standard of care at our institution recommends ECMO as a rescue therapy for ARDS when a patient requires extensive invasive mechanical ventilation that exceeds the recommendations of the respective clinical guidelines [8]. Based on the institutional protocols, ECMO was considered when *p*_a_O_2_/*F*_i_O_2_ remained <100 mmHg after 6 hours of conventional ARDS therapy and at least one of the following additional criteria was present: respiratory acidosis with a pH <7.2, tidal volumes >6 ml/kg ideal body weight (IBW) or a plateau pressure ≥35 mbar to ensure oxygenation, invasive ventilation for less than 7 days, ventilation-induced pneumothorax or mediastinal emphysema, and a Murray lung injury score ≥3.

VV-ECMO placement was performed under echocardiographic guidance [9]. Cannulation was performed as follows: a 23 Fr to 25 Fr draining cannula was inserted into the femoral vein and advanced into the vena cava inferior and a 17 to 19 Fr return cannula was inserted into the right internal jugular vein and advanced *n* the vena cava superior. Over the study period, three different types of centrifugal pumps were used, namely, iLA activve® (Xenios/Novalung®, Heilbronn, Germany), Rotaflow, and Cardiohelp (Getinge/Maquet®, Rastatt, Germany).

### 2.3. Ventilation Strategy during ECMO Therapy

During the first 48 hours after VV-ECMO was initiated, all patients received sedation targeting a Richmond Agitation and Sedation Scale of −5. An ultra-low tidal volume ventilation was the main therapeutic target after VV-ECMO placement per institutional protocol. Using a pressure-controlled ventilation mode, we aimed for a tidal volume of 3.5 ml/kg ideal body weight in all patients. Ideally, this was achieved using an inspiratory plateau pressure ≤25 mbar and a positive end-expiratory pressure arbitrarily set to 15 mbar.

### 2.4. Echocardiographic Measurements

In a subset of patients, we analyzed the available echocardiographic images. For this, the picture archiving and communication system (PACS) of the University Hospital Tübingen was searched for echocardiographic images from both cohorts. The respective images were further analyzed, if recorded within 24 hours after ECMO implantation. All measurements were performed offline according to the current *American Society of Echocardiography* recommendation on a vendor-independent platform (Philips IntelliSpace Cardiovascular Ver. 3.2 system, Philips Medical Systems Nederland B. V., Best, The Netherlands) by an echocardiography-certified critical care physician. As a marker of right-ventricular function, we determined the right-ventricular fractional area-change (RVFAC). As a marker for systolic left-ventricular function, we measured the left ventricular ejection fraction based on the Simpson method.

### 2.5. Data Collection and Matching

For propensity score matching, we screened all patients admitted to our intensive care unit (ICU) from December 2014 to May 2018. Next, we selected patients who received the ICD-10 code J80 (*acute respiratory distress syndrome in the adult*). Only patients who underwent VV-ECMO treatment were included. Based on the data of the clinical information systems, a database containing relevant patient information, including age, sex, date of VV-ECMO initiation and VV-ECMO duration, and the Sequential Organ Failure Assessment (SOFA), Simplified Acute Physiology Score II (SAPS II), and Acute Physiology and Chronic Health Evaluation (APACHE) scores, was generated. Comorbidities were also recorded from the patient records (e.g., chronic obstructive pulmonary disease, nicotine use, history of lung disease, coronary artery disease, and diabetes mellitus) as well as complications during ECMO treatment if they were explicitly mentioned in the patient records.

To compare survivors to nonsurvivors, a 1 : 1 matching was performed using the propensity score matching method based on the following variables that were expected to be associated with the ICU outcome: age, ARDS severity based on the Berlin definition [1], *p*_a_O_2_/*F*_i_O_2_ prior to VV-ECMO, and SAPSII on the day of ECMO implantation. Matching was performed in Microsoft Excel® using the XLSTAT® software (Addinsoft Inc.) based on the Mahalanobis distance best match algorithm as previously described [10].

### 2.6. Outcome and Driving Pressure

To analyze the mechanics of the respiratory system, we extracted the ventilator measurements and settings from the *Patient Data Management System* (PDMS). We integrated the following measurements: plateau pressure (*P*_plat_), positive end-expiratory pressure (PEEP), breathing rate, tidal volume corrected to ideal body weight (IBW; *V*_t/IBW_), and mean airway pressure (*P*_mean_). All patients underwent controlled mechanical ventilation, and thus we used the following formula to calculate the driving pressure (Δ*P*): Δ*P* = *P*_plat_−PEEP. To identify a ventilatory parameter eligible as an early marker for disease progression after ECMO initiation, we assessed the values 30 min prior to VV-ECMO initiation and at 12 hours after initiation of treatment. The change in driving pressure was calculated as follows: % Δ*P*_change_ = [(Δ*P*_before EMCO_–Δ*P*_after EMCO_)/Δ*P*_after ECMO_] *×* (−100).

### 2.7. Statistical Analysis

Continuous variables are expressed as the mean ± standard deviation and were compared using the Mann–Whitney *U* test. Categorical variables, such as the preadmission demographics, were compared using Fisher's exact test to assess the association of different variables with mortality logistic regression analyses that were performed. The discriminatory power of the early driving pressure change for predicting mortality was assessed by calculating the area under the receiver operating characteristic (ROC) curve (AUC). A cut-off value was calculated based on the Youden index. All statistical analyses for this study were performed in Prism 8 (GraphPad Software Inc.) and in JMP 14.2 (SAS Institute Inc., Cary, NC, USA). The *p* values are two-tailed, and values <0.05 are considered statistically significant.

## 3. Results

### 3.1. Patient Selection and Characteristics

**Figure 1:**
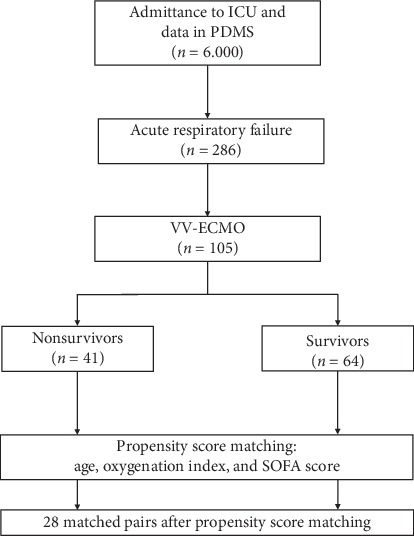
Patient selection and matching strategy.

The matching process is depicted in Figure 1. From the 105 patients who underwent VV-ECMO, we defined two groups of matched patients (*n* = 28 per group). The groups did not differ in their baseline characteristics (age, sex, height, weight, and body mass index) or comorbidities (Table 1). Based on the analysis of demographics and comorbidities, we concluded that the matching process had identified a homogenous study cohort.

### 3.2. ICU Variables at Admission

The study groups did not differ in their major critical illness prediction scores, the duration of mechanical ventilation, length of ICU stay, *P*_a_O_2_/*F*_i_O_2_ ratio before ECMO, renal replacement therapy, fluid balance, or duration of ECMO therapy (Table 2). Hemodynamic variables such as mean arterial pressure and heart rate did not differ between our study cohorts (Table 2). Overall, these results suggest that the matched nonsurvivors and survivors of ECMO therapy were comparable regarding their demographic variables, in terms of their ARDS severity and subsequent organ failure.

### 3.3. ARDS Etiologies

Next, we investigated whether the nonsurvivors and survivors diverged in the etiologies of ARDS. We categorized the suspected etiologies of ARDS based on the patients' medical records. Our study cohort included many patients who developed primary ARDS because of pneumonia (40 of 56 patients, 66%). A difference was found between the survivors and nonsurvivors regarding the microbiological profile causing pneumonia; the survivors of ECMO developed ARDS mainly because of a viral pneumonia (*p*=0.03) (Table 3). Extrapulmonary bacterial infections were an uncommon finding to trigger ARDS in our study cohort. Other causes of ARDS, such as thorax trauma and pancreatitis, were rare causes of ARDS in both groups.

### 3.4. Echocardiographic Findings

As patients outcome could have been influenced by acute or chronic dysfunction in myocardial contractility, we analyzed echocardiographic images taken within 24 hours after VV-ECMO. Unfortunately, we could only retrieve sufficient cardiac images in 23 patients (12 survivors and 11 nonsurvivors). Regarding these patients, right and left ventricular function did not differ between the two study groups (Supplementary 1).

### 3.5. Invasive Ventilation Variables

We investigated whether mechanical ventilation as a surrogate for the extent of respiratory mechanics varied between the groups. For this analysis, we compared the ventilatory parameters within 30 min before ECMO initiation and at 12 h after the beginning of ECMO (Table 4). ECMO setting in the available data set did not differ between groups. All patients received pressure-controlled ventilation. The *P*_plat_ before ECMO was not different between the survivors and nonsurvivors, but the survivors trended towards a higher *P*_plat_ before ECMO. The *P*_mean_, PEEP, and the ventilatory rate were identical between the survivors and nonsurvivors both before and after 12 hours of ECMO treatment. Both study groups received ventilation with low tidal volumes before ECMO (*V*_t/IBW_ nonsurvivors 5.7 ± 2.3 ml/kg/IBW; survivors 6.3 ± 1.6 ml/kg/ideal body weight; *p*=0.79). After initiation of ECMO, the tidal volumes decreased, with no significant difference between the groups (*V*_t/IBW_ nonsurvivors 3.3 ± 4.8 ml/kg/ideal body weight; survivors 3.7 ± 1.8 ml/kg/ideal body weight; *p*=0.79).

**Figure 2:**
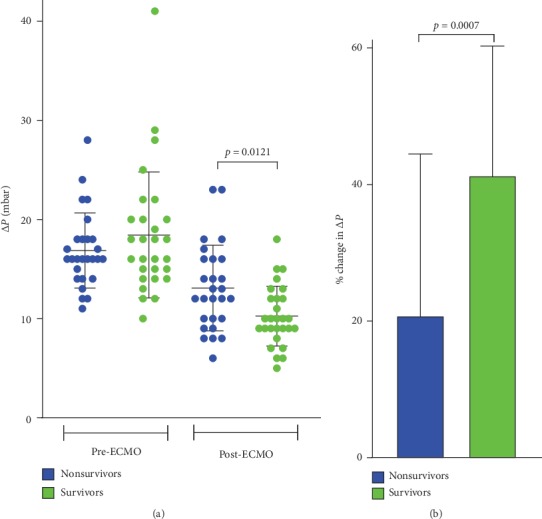
Driving pressures in the survivors and nonsurvivors. (a) Driving pressures (Δ*P*; mbar) in the nonsurviving and surviving patients receiving extracorporeal membrane oxygenation before and after initiation of therapy. (b) Percentage of Δ*P* changes before and after ECMO initiation (mean ± SD; *n* = 28 per group).

Since ΔP has been reported recently as an outcome-predicting variable in ARDS [11] and ECMO [12, 13], next we analyzed whether the ventilation driving pressure distinguished the survivors from the nonsurvivors at an early time point after initiation of therapy. As shown in Table 4, in both cohorts, ΔP was greater than 15 mbar (nonsurvivors 16.8 ± 3.8 mbar; survivors 18.4 ± 6.3 mbar; *p*=0.5) and static respiratory compliance was similar between groups before ECMO. In correspondence with the other invasive ventilation parameters, ΔP was decreased in both groups at 12 hours after starting ECMO (Table 4 and Figure 2(a)). Yet, survivors had significantly higher static compliance values, translating into significantly lower driving pressure than the nonsurvivors at this time point (nonsurvivors 13.0 ± 4.3 mbar; survivors 10.3 ± 3.0 mbar; *p*=0.01). Next, the change in ΔP was calculated. This analysis revealed that the driving pressure was reduced by 41.3% at 12 hours after starting ECMO in the survivors versus only 20.8% in the nonsurvivors (*p* < 0.01). Taken together, our data show that the survivors exceed the nonsurvivors in terms of changes in ΔP changes by almost 2-fold.

### 3.6. Logistic Regression Analysis of Driving Pressure Changes

**Figure 3:**
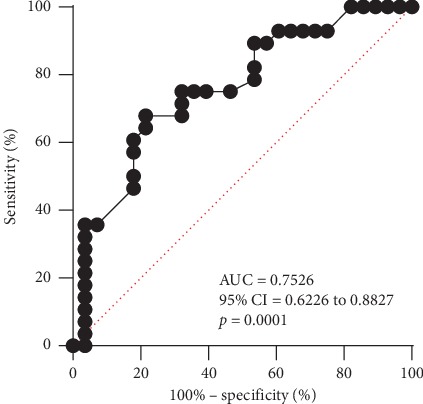
Receiver-operating characteristic curve (ROC) for relative driving pressure change. ROC analysis for the relative change in driving pressure and survival of ECMO therapy; area under the curve (AUC) 0.75265 (chi^2^ = 11.27336; *p*=0.0008).

Logistic regression analysis was performed to assess the impact of known markers of ICU survival on mortality (Table 5). As shown in Table 5, only infectious versus noninfectious causes of ARDS and Δ*P*-based parameters (Δ*P* at 12 hours, absolute and relative Δ*P* change) were significant predictors of VV-ECMO survival. ROC analysis displayed a significant discrimination with an AUC of 0.75265 (95% CI 0.6226 to 0.8827, *p*=0.0001, Figure 3). The optimum cut-off point to discriminate between survivors and nonsurvivors was a 33% change in driving pressure within the first 12 hours with a sensitivity of 78% and a specificity of 67.9%.

As the different Δ*P*-based parameters showed a strong correlation with each other in a multivariable correlation analysis, we chose the relative Δ*P* change (that had the lowest *p* value in the univariable analysis) for the multivariable approach. In the multivariable approach, again, the etiology of ARDS and the relative DP change were significant predictors of VV-ECMO outcome (Table 5). The ROC analysis showed a high significant discrimination with an AUC of 0.8638 (95% CI 0.7553 to 0.9724, *p* < 0.0001).

## 4. Discussion

Using propensity score matching, we identified 28 pairs of surviving and nonsurviving ARDS patients who underwent VV-ECMO. To identify outcome-predicting parameters, we focused on mechanical ventilation parameters collected before and 12 hours after VV-ECMO initiation. Our main finding was that despite the use of low tidal volume ventilation in both groups, the survivors exhibited a change in Δ*P* of 40%, whereas in the nonsurvivors, Δ*P* was only reduced by 20%. Logistic regression analysis identified the driving pressure change and the etiology of the ARDS as significant predictors for ECMO survival. This result indicates that driving pressure reduction can be used as an early diagnostic parameter for risk stratification to assess whether patients may benefit from ECMO.

Δ*P* results from the subtraction of PEEP from plateau pressure [11, 14]. Physiologically, Δ*P* represents the tidal volume corrected for a patient's compliance with the respiratory system [15]. The connection between Δ*P* and ARDS-related mortality was first recognized in 2002, when a prospective observational study found that Δ*P* was the only ventilation variable that differed between survivors and nonsurvivors [14]. This finding was confirmed in a large retrospective analysis of more than 3500 patients from various randomized trials. Here, Δ*P* correlated best with survival of ARDS patients. Δ*P* even predicted ARDS case-related mortality for patients who received low tidal volume ventilation [11]. The study identified 15 cm H_2_O for Δ*P* as a threshold for a positive outcome. In our cohort, Δ*P* before ECMO was greater than 15 cmH_2_O in both groups but was decreased in both groups 12 hours after VV-ECMO initiation. This finding was is in line with other studies that found that Δ*P* values correlated with survival after ECMO in general [12, 13]. In our patients, Δ*P* changes were significantly different between the survivors and nonsurvivors at a very early time point after ECMO (12 hours), with the change reduction in Δ*P* almost being double in the survivors compared to that in the nonsurvivors (Figure 2(b)). Taken together, our results indicate that changes in Δ*P* have diagnostic value for assessment of lung injury severity in cases in which *P*_a_O_2_/*F*_i_O_2_ cannot be used because ECMO interferes with the read-out.

To determine Δ*P* in the present study, we had to rely on the values measured automatically by the ventilator. *P*_plat_ and PEEP were not measured through occlusion, which usually is required to get accurate measurements [16]. Thus, the values determined through the medical record in this retrospective study could differ from the absolute values measured through occlusion of the respiratory system. At the time of measurement, both groups were deeply sedated (goal for Richmond Agitation and Sedation scale −5), which made spontaneously breathing efforts unlikely and limits artefacts in the measurements. Furthermore, both groups—survivors and nonsurvivors—underwent the same treatment protocol. Thus, values used to determine Δ*P* might not reflect the absolute pressure values determined by occlusion, but the difference observed remains a valid observation.

In our cohort, the predominant underlying cause of ARDS was pneumonia. In terms of the microbiological agent, we found a trend towards more bacterial pneumonia in the nonsurvivors. In contrast, the surviving patients developed significantly more ARDS based on viral pneumonia. This result is in line with that of a retrospective analysis by Schmidt et al. who found that ECMO for severe asthma and viral pneumonia was independently associated with hospital survival [17]. The reason for this finding is unclear; however, we can imagine that the inflammatory responses to bacterial and viral pneumonia differ fundamentally. Indeed, Calfee et al. described different ARDS subphenotypes [18], with different clinical characteristics and outcomes.

The current clinical guidelines for treatment and mechanical ventilation of ARDS patients focus on plateau pressure targets and tidal volumes corrected to the ideal body weight [8]. ECMO is indicated, when mechanical ventilation comes with the price of an excessively high plateau pressure, despite optimal standard care [19]. ECMO facilitates deescalation of mechanical ventilation, but the ventilation strategy for patients undergoing ECMO for ARDS treatment remains a subject of debate. No randomized controlled trial has evaluated mechanical ventilation in patients undergoing ECMO despite the increasing numbers of ECMO cases over the last few years [4]. Some authors refer to this approach as “lung rest” [19] or “ultraprotective ventilation” [20], which leads to a reduction of proinflammatory cytokines in the lung [21]. The rationale behind lung rest is that even tidal volumes of 6 ml/kg/IBW can lead to regional pulmonary overdistension. In ECMO patients, most centers globally use ECMO therapy to facilitate “lung rest” [22], and several authors recommend lung rest ventilation as a strategy of choice for patients undergoing ECMO [23]. Currently, at our institution, we establish an ECMO circuit in patients who cannot be mechanically ventilated within lung-protective limits. Once ECMO is established, we also follow a lung rest concept to reduce alveolar strain. As shown in Table 4, the tidal volumes decreased at 12 hours after ECMO to below 4 ml/kg/IBW; simultaneously, the PEEP remained high for lung recruitment based on the empirical PEEP-*F*_i_O_2_ strategy [24] as recommended by our current published practice guidelines for ARDS [25]. All ARDS patients in our ICU adopt a prone position for at least 16 hours per day until compliance improves regardless of use of ECMO [26]. Regarding the ventilator settings, the mean plateau pressure also decreased in both groups (Table 4) to below 30 cm H2O, albeit to a lesser extent in the nonsurvivors. Taken together, the very early driving pressure changes seen within 12 hours most lightly reflect reductions in overdistensions. Therefore, this parameter might identify patients in whom ventilator settings should be further deescalated.

To enhance the comparability between the two groups, we used a propensity score matching approach similar to that of other studies [27]. Using this approach, almost half of the 105 ECMO patients treated in our institution since 2014 could not be matched in either group, which might have resulted in selection bias. However, although we analyzed a smaller set of patients than other studies [12, 13], we independently found that the driving pressure seemed to be the respiratory system mechanics' variable that was associated with survival after ECMO, indicating the validity of our findings. Although the change in Δ*P* significantly separated the two groups from each other, we cannot prove that patient survival was a consequence of changes in ΔP due to the design of this study. In our opinion, a prospective randomized control trial is required to define whether Δ*P* is a therapeutic target in ARDS patients.

## 5. Conclusion

In summary, we report that patients surviving ECMO undergo a drastic decrease in driving pressure within the first 12 hours of ECMO, whereas in nonsurvivors, driving pressure changes are strongly attenuated. Future research needs to clarify whether strategies to adjust ventilator settings in ECMO patients based on driving pressure calculations may improve patient outcomes.

## Figures and Tables

**Table 1:** Demographic data and comorbidities.

	Nonsurvivors (*n* = 28)	Survivors (*n* = 28)	*p* values
*Demographic data*			
Age, yr (mean ± SD)	54 ± 10	53 ± 15	0.7727
Male sex, no. (%)	20 (71.4%)	21 (75.0%)	>0.99
Height (cm)	173 ± 10	175 ± 9	0.4469
Weight (kg)	91 ± 30	91 ± 22	0.5050
Body Mass Index (kg/m^2^)	30 ± 8	30 ± 6.0	0.6993

*Comorbidities*, *no. (%)*			
Diabetes mellitus	6 (21.4%)	7 (25.0%)	>0.99
Nicotine use	10 (35.7%)	10 (35.7%)	>0.99
Chronic obstructive pulmonary disease	4 (14.3%)	3 (10.7%)	>0.99
Chronic renal failure	3 (10.7%)	4 (14.3%)	>0.99
Autoimmune disease	4 (14.3%)	4 (14.3%)	>0.99
Arterial hypertension	6 (21.4%)	12 (43.0%)	0.1516
History of malignancy	3 (10.7%)	6 (21.4%)	0.4688
History of substance abuse (incl. alcohol)	6 (21.4%)	5 (17.9%)	>0.99
Peripheral atherosclerotic disease	4 (14.3%)	4 (14.3%)	>0.99
Coronary artery disease	5 (17.8%)	2 (7.1%)	0.4216
Neurological disease	6 (21.4%)	7 (25.0%)	>0.99

**Table 2:** ICU patient variables.

	Nonsurvivors (*n* = 28)	Survivors (*n* = 28)	*p* values
Median hours of mechanical ventilation (h)	442 (188–800)	523 (327–765)	*0.4891*
SAPS II (mean ± SD)	44.8 ± 10.7	44.9 ± 16.0	*0.7727*
APACHE II (mean ± SD)	25.3 ± 7.7	26.8 ± 13.4	*0.9529*
SOFA	11.9 ± 2.8	10.7 ± 2.7	*0.1262*
Renal replacement therapy in ICU	17 (60.7%)	15 (53.6%)	*0.7875*
Median days length of ICU stay (interquartile range)	18.5 (9–36.75)	24 (15.5–32.00)	*0.2515*
Median days duration of ECMO (interquartile range)	16 (7–28.75)	18 (8.5–24.0)	*0.9838*

*Variables before ECMO implantation*			
Median days of invasive ventilation (interquartile range)	1.0 (1–5)	1 (0–3)	*0.2701*
Median *p*O_2_/*F*iO_2_ ratio (interquartile range) (mmHg)	62 (48–89)	67 (61–90)	*0.2223*
Median *p*CO_2_ (interquartile range) (mmHg)	51 (47–60)	51 (47–57)	*0.5896*

*Fluid balance 24 hours after ICU admission*			
Median fluid balance (interquartile range) (ml)	2125 (0–4017)	1375 (131–3334)	*0.9795*

*Hemodynamic, respiratory, and biochemical variables*			
Median norepinephrine (interquartile range) (*µ*g/kg/min)			
Before ECMO	0.14 (0.00–0.33)	0.1 (0–0.33)	*0.8553*
12 h after ECMO	0.08 (0.00–0.24)	0.09 (0.02–0.20)	*0.8827*
Median serum lactate (interquartile range) (mmol/l)			
Before ECMO	2.0 (1.2–3.6)	1.4 (0.9–3.0)	*0.2758*
12 h after ECMO	1.9 (1.2–3.4)	1.5 (1.0–2.6)	*0.2127*
Heart rate (beats/minute)			
Before ECMO	106 ± 21	98 ± 20	*0.3419*
12 h after ECMO	91 ± 21	88 ± 18	*0.7402*
Mean arterial pressure (mmHg)			
Before ECMO	78 ± 13	80 ± 15	*0.4051*
12 h after ECMO	74 ± 11	72 ± 9	*0.3815*

**Table 3:** Etiologies of acute respiratory distress syndrome.

	All patients (*n* = 56)	Nonsurvivors (*n* = 28)	Survivors (*n* = 28)	*p* values
Pneumonia	37	16	21	0.4219
Bacterial infection	16	10	6	0.3753
Viral infection	21	6	15	***0.0261***
Extrapulmonary bacterial infection	4	3	1	0.6110
Aspiration of gastric content	7	5	2	0.4216
Thorax trauma	2	1	1	>0.9999
Morbus Wegener	2	1	1	>0.9999
Acute pancreatitis	4	2	2	>0.9999

**Table 4:** Ventilator variables.

	Nonsurvivors	Survivors	*p* values
*VV-setting ECMO*	(*n* = 15)	(*n* = 17)	
Blood flow (l/min)	4.0	4.6	*0.0799*
Rotation per minute (rpm)	4160	3427	*0.1163*
Sweep gas flow (l/min)	3.967	3.882	*0.8647*

*Plateau pressure (mbar)*	(*n* = 28)	(*n* = 28)	
Before ECMO	32.2 ± 4.8	35.0 ± 6.7	*0.0998*
12 h after ECMO	27.1 ± 4.1	25.6 ± 3.2	*0.1496*

*Positive end-expiratory pressure (mbar)*	(*n* = 28)	(*n* = 28)	
Before ECMO	15.0 ± 3.4	15.4 ± 3.1	*0.7798*
12 h after ECMO	14.0 ± 2.8	15.3 ± 2.7	*0.1049*

*Mean airway pressure (mbar)*	(*n* = 28)	(*n* = 28)	
Before ECMO	22.7 ± 4.8	22.0 ± 4.1	*0.7309*
12 h after ECMO	20.2 ± 2.5	19.2 ± 3.3	*0.2278*

*Respiratory rate (per minute)*	(*n* = 28)	(*n* = 28)	
Before ECMO	21 ± 5	23 ± 8	*0.7076*
12 h after ECMO	15 ± 6	14 ± 5	*0.4264*

*Tidal volumes/ideal body weight (ml/kg)*	(*n* = 28)	(*n* = 28)	
Before ECMO	5.7 ± 2.3	6.3 ± 1.6	*0.7875*
12 h after ECMO	3.3 ± 4.8	3.7 ± 1.8	*0.5783*

*Static compliance (ml/mbar)*	(*n* = 28)	(*n* = 28)	
Before ECMO	28 ± 11	25 ± 10	*0.4976*
12 h after ECMO	22 ± 13	31 ± 18	***0.0447***

*Driving pressure (mbar)*	(*n* = 28)	(*n* = 28)	
Before ECMO	16.9 ± 3.8	18.4 ± 6.3	*0.4976*
12 h after ECMO	13.0 ± 4.3	10.3 ± 3.0	***0.0121***

**Table 5:** Factors associated with mortality.

	Univariate logistic regression		Multivariate logistic regression	
	Odds ratio (95% CI)	*p* value	Odds ratio (95% CI)	*p* value
*paO*_*2*_ */FiO*_*2*_ *ratio before VV-ECMO*	0.988 (0.97 to 1.01)	0.2079		

*Serum lactate*				
Before ECMO	1.12 (0.89 to 1.41)	0.3463		
12 h after ECMO	1.12 (0.92 to 1.37)	0.2529		

*Etiology of ARDS*				
Bacterial infection versus noninfectious	0.19 (0.04 to 0.74)	0.0338	0.09 (0.01 to 0.97)	***0.0099***
Viral infection versus noninfectious	0.19 (0.04 to 0.74)	**0.0161**	0.08 (0.01 to 0.42)	***0.0021***
Bacterial versus viral infection	1.02 (0.24 to 4.22)	0.9830		

*Driving pressure*				
Before ECMO	0.94 (0.84 to 1.05)	0.2770		
12 h after ECMO	1.25 (1.05 to 1.48)	**0.0129**		
Absolute change	0.82 (0.71 to 0.94)	**0.0048**		
Relative change (per 1% decrease)	0.96 (0.93 to 0.99)	**0.0033**	**0.94 (0.90 to 0.97)**	***<0.0001***

## Data Availability

All data included in this study are available from the corresponding author upon reasonable request.

## Supplementary Materials

Supplementary Materials Echocardiographic parameters of right and left ventricular function.
