# Supplementary material for: Early Driving Pressure Changes Predict Outcomes during Venovenous Extracorporeal Membrane Oxygenation for Acute Respiratory Distress Syndrome
Source: Crit Care Res Pract. 2020 Mar 7;2020:6958152. doi: 10.1155/2020/6958152 (PMC7085355; doi:10.1155/2020/6958152)
Supplement: Supplementary Materials — Echocardiographic parameters of right and left ventricular function. [file 6958152.f1.pdf]

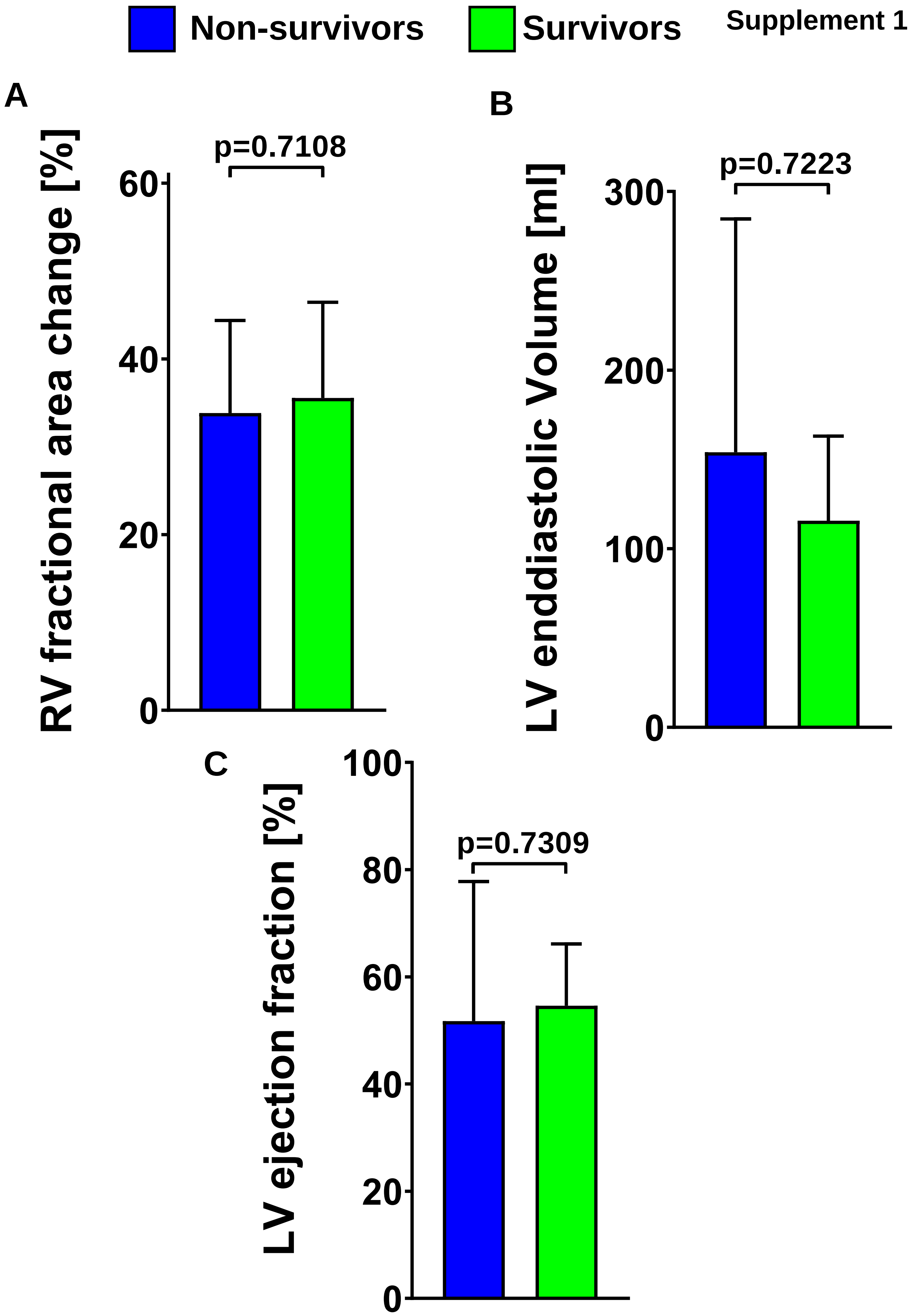
Non-survivors
Survivors
Supplement 1
A
B
C
p=0.7108
RV fractional area change [%]
60
40
20
0
p=0.7223
LV enddiastolic Volume [ml]
300
200
100
0
p=0.7309
LV ejection fraction [%]
100
80
60
40
20
0
